# Supplementary material for: What are the biopsychosocial risk factors associated with pain in postpartum runners? Development of a clinical decision tool
Source: PLoS One. 2021 Aug 12;16(8):e0255383. doi: 10.1371/journal.pone.0255383 (PMC8360599; doi:10.1371/journal.pone.0255383)
Supplement: S1 File — (DOCX) [file pone.0255383.s001.docx]

S1 file: Qualtrics survey seen by participants

Postpartum runner survey


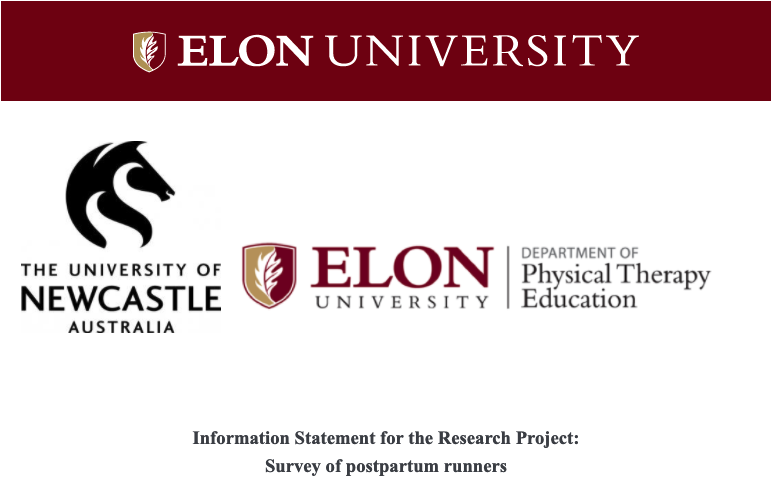


You are invited to participate in the research project identified above which is being conducted by Shefali Christopher, PT, DPT, SCS, LAT, ATC, assistant professor at Elon University and PhD candidate from the School of Health Sciences at the University of Newcastle Australia, A/Prof Suzanne Snodgrass and Prof Chad Cook. The research is part of Shefali Christopher’s PhD studies at the University of Newcastle, supervised by Suzanne Snodgrass from the School of Health Sciences at University of Newcastle and Prof Chad Cook from Duke University.  
 Why is the research being done?
 Guidelines for returning to running after having a baby are sparse. Many social media outlets have conflicting information and it can be difficult for postpartum women to understand how to get back to running safely. What we do know is that a large number of postpartum women runners have difficulties when running (pain, incontinence, etc). The aim of this study is to understand the habits of postpartum runners as well as measure other variables that affect their running.
 Who can participate in the research?
 Any woman 18 yrs or older, who is trying to run at least one time per week on average, and has a child 36 months (3 years) or younger is eligible to participate in this survey.
 The survey has been developed in collaboration with experts in postpartum running and with postpartum runners. 
    What would you be asked to do? If you agree to participate, you will be asked to complete an anonymous online questionnaire that asks about your current running routines and includes questions about you (age, etc.), questions that relate to postpartum depression, questions related to incontinence, and if you have pain, questions related to your pain when running. This survey will be open for 1 year or until we have collected 5,000 responses.
 What choice do you have?
 Participation in this research is entirely your choice. Only those people who give their consent will be included in the project. Whether or not you decide to participate, your decision will not disadvantage you. If you do decide to participate, you may withdraw from the project at any time prior to submitting your completed questionnaire. Please note that due to the anonymous nature of the questionnaire, you will not be able to withdraw your response after it has been submitted.
  
 How much time will it take?
 The questionnaire takes approximately fifteen minutes to complete if you do experience pain, and about five minutes if you are a postpartum runner with no pain.
 While there are no anticipated benefits to you personally in participating in this research, the findings will contribute to the available literature on the subject and help clinicians worldwide have a better understanding of postpartum runners with and without pain.

How will your privacy be protected?
 Your questionnaire responses are anonymous. The questionnaire responses will be stored on a password-protected server through Qualtrics software. This company is a common vendor used for survey research and has significant data protection policies in place. Please see the Qualtrics security statement here: http://www.qualtrics.com/security-statement/. Following the data collection period, the data will be downloaded from the Qualtrics server and securely stored on Shefali Christopher's encrypted and password-protected laptop computer, with a back-up copy securely stored on the University of Newcastle's secure cloud server. Your data will be retained for a minimum of 5 years as per University of Newcastle policy provisions and destroyed via deletion in accordance with University of Newcastle and Elon University policies. To the extent allowed by law, we limit the viewing of your personal information to people who must review it. The Institutional Review Board (IRB), Elon University and the Human Research Ethics Committee, University of Newcastle (Australia), and other representatives of these organizations may inspect and copy your information.
  
 How will the information collected be used?
 The collected data will contribute to Shefali Christopher’s Ph.D. thesis and may be presented in peer-reviewed publications or conferences. You can access a copy of the published report by visiting this webpage: https://www.elon.edu/e/directory/profile.html?user=schristopher3 after July 2020. Individual participants will not be named or identified in any reports arising from the project.
  
 What do you need to do to participate?
 Please read this Information Statement and be sure you understand its contents before you consent to participate. If there is anything you do not understand, or you have questions, please contact the research team. If you would like to participate, please complete the questionnaire by clicking on this link: (Survey of postpartum runners). Completion and submission of this online questionnaire will be taken as your consent to participate.   Will you be paid for participating?
 Three $25 Amazon gift cards will be given away each month to participants who complete the questionnaire and choose to enter the drawing. A link will be provided at the end of the survey in order to provide your contact details separate to the questionnaire (your questionnaire responses will remain anonymous).
  
 Further information
 If you would like further information, please contact one of the members of the research team below:
  
 Shefali Christopher
 Assistant Professor, Elon University
 Tel: +1-336-278-6416; Email: schristopher3@elon.edu
  
 Associate Professor Suzanne Snodgrass
 Associate Professor, University of Newcastle, Australia
 Tel: +61 2 4921 2089; Email: Suzanne.Snodgrass@newcastle.edu.au
  
 Professor Chad Cook
 Professor, Duke University
 Tel: +1-919-684-8905; Email: Chad.Cook@duke.edu

 Complaints about this research
 This project has been approved by the University’s Newcastle's Human Research Ethics Committee, Approval No H-2019-0118 and Elon University's Institutional Review Board (Protocol #19-222)
 Should you have concerns about your rights as a participant in this research, or you have a complaint about the manner in which the research is conducted, it may be given to the researcher, or, if an independent person is preferred, to Stephen Bailey Elon IRB chair, telephone (336) 278-6346 or e-mail baileys@elon.edu.. You may also contact the Human Research Ethics Officer, Research Services, NIER Precinct, The University of Newcastle, University Drive, Callaghan NSW 2308, Australia, telephone +61 (2) 4921 6333, email Human-Ethics@newcastle.edu.au, email Human-Ethics@newcastle.edu.au.
If you would like to complete the questionnaire, please click on the arrow below.

Please fill out the following information: 

Do you have a child 36 months (3 years) or younger?

- Yes
- No

Display This Question:

If Please fill out the following information:  Do you have a child 36 months (3 years) or younger? = No

Thank you for your participation. You have indicated that you do not have a child under the age of three. We are currently only including postpartum runners who have a child under three and are trying to run a least 1x/week on average unless they are limited by pain. If you feel you have received this message in error, please contact schristopher3@elon.edu

Skip To: End of Survey If Thank you for your participation. You have indicated that you do not have a child under the age o... Is Displayed

Are you currently pregnant?

- Yes
- No

Display This Question:

If Are you currently pregnant? = Yes

Thank you for your participation. You have indicated that you are currently pregnant. We are only including postpartum runners at this time. If you feel you have received this message in error, please contact schristopher3@elon.edu

Skip To: End of Survey If Thank you for your participation. You have indicated that you are currently pregnant. We are only... Is Displayed

How many times did you run in the last four weeks?

- 0
- 1
- 2
- 3
- 4
- 5
- 6
- 7
- 8
- 9
- 10
- >10

Display This Question:

If How many times did you run in the last four weeks? = 1

Or How many times did you run in the last four weeks? = 2

Or How many times did you run in the last four weeks? = 3

Or How many times did you run in the last four weeks? = 0

Why are you currently unable to run at least one time per week?

- My pain limits me
- I cannot find the time
- Other- Please Specify: ________________________________________________

Display This Question:

If Why are you currently unable to run at least one time per week? = I cannot find the time

Thank you for your participation. You have indicated that you are currently not running at least one time a week. We are currently only including postpartum runners who are trying to run a least 1x/week on average unless they are limited by pain. If you feel you have received this message in error, please contact schristopher3@elon.edu.

Skip To: End of Survey If Thank you for your participation. You have indicated that you are currently not running at least... Is Displayed

End of Block: Default Question Block

Start of Block: Running info

**The following questions are about your running habits.**
 
  How many total miles/ kilometers did you average in the past 7 days?

- Miles ________________________________________________
- Kilometers ________________________________________________

Has your running mileage changed due to COVID-19?

- Yes, it has increased
- Yes, it has decreased
- No change in amount of running

When did you start running after having a baby? (in weeks)

________________________________________________________________

What type of runner are you **currently**?

- Novice (I am new to running)
- Recreational (I run for exercise, race for fun)
- Competitive (I have a training plan and race to achieve personal best time)
- Elite (I place in the top 10% of my age group at races)
- Other- Please Specify: ________________________________________________

Do you currently run with a stroller/pram?

- Yes (1)
- No (2)

End of Block: Running info

Start of Block: Pain

**The following questions ask you about your current pain when running.**
  Do you currently have any pain when running?

- Yes
- No

End of Block: Pain

Start of Block: Depression

As you have recently had a baby, we would like to know how you are feeling. Please check the answer that comes closest to how you have felt IN THE PAST 7 DAYS, not just how you feel today.
 
 
In the past 7 days:

I have been able to laugh and see the funny side of things

- As much as I always could
- Not quite so much now
- Definitely not so much now
- Not at all

I have looked forward with enjoyment to things

- As much as I ever did
- Rather less than I used to
- Definitely less than I used to
- Hardly at all

I have blamed myself unnecessarily when things went wrong

- Yes, most of the time
- Yes, some of the time
- Not very often
- No, never

I have been anxious or worried for no good reason

- No, not at all
- Hardly ever
- Yes, sometimes
- Yes, very often

I have felt scared or panicky for no very good reason

- Yes, quite a lot
- Yes, sometimes
- No, not much
- No, not at all

Things have been getting on top of me

- Yes, most of the time I haven't been able to cope at all
- Yes, sometimes I haven't been coping as well as usual
- No, most of the time I have coped quite well
- No, I have been coping as well as ever

I have been so unhappy that I have had difficulty sleeping

- Yes, most of the time
- Yes, sometimes
- Not very often
- No, not at all

I have felt sad or miserable

- Yes, most of the time
- Yes, quite often
- Not very often
- No, not at all

I have been so unhappy that I have been crying

- Yes, most of the time
- Yes, quite often
- Only occasionally
- No, never

The thought of harming myself has occurred to me

- Yes, quite often
- Sometimes
- Hardly ever
- Never

Display This Question:

If The thought of harming myself has occured to me = Yes, quite often

Or The thought of harming myself has occured to me = Sometimes

Thank you for your response. If you are feeling sad, anxious, and depressed or have plans to harm yourself please reach out to an emergency medical provider immediately.   This is a worldwide survey and the following information will be displayed   You can also reach out to a hotline for support with depression below: Argentina: +5402234930430 Australia: 131114 Austria: 017133374 Belgium: 106 Bosnia & Herzegovina: 080 05 03 05 Botswana: 3911270 Brazil: 188 for the CVV National Association Canada: 5147234000 (Montreal); 18662773553 (outside Montreal) Croatia: 014833888 Denmark: +4570201201 Egypt: 7621602 Estonia: 3726558088; in Russian 3726555688 Finland: 010 195 202 France: 0145394000 Germany: 08001810771 Holland: 09000767 Hong Kong: +852 2382 0000 Hungary: 116123 India: 8888817666 Ireland: +4408457909090 Italy: 800860022 Japan: +810352869090 Mexico: 5255102550 New Zealand: 0800543354 Norway: +4781533300 Philippines: 028969191 Poland: 5270000 Portugal: 21 854 07 40/8 . 96 898 21 50 Russia: 0078202577577 Spain: 914590050 South Africa: 0514445691 Sweden: 46317112400 Switzerland: 143 United Kingdom: 08457909090 USA: 18002738255 Veterans' Crisis Line: 1 800 273 8255/ text 838255 (https://ibpf.org/resource/list-international-suicide-hotlines)

End of Block: Depression

Start of Block: Delivery, fatigue, sleep, other

**The following questions ask about your current level of fatigue.**

   In the last **two weeks** have you felt fatigued?

- Yes
- No

Display This Question:

If The following questions ask about your current level of fatigue.   In the last two weeks have you... = Yes

Please answer the following questions about your fatigue:

|  |  |
| --- | --- |
| Feel tired in the morning | ▼ Rarely ... Often |
| Feel exhausted (excluding after exercising) | ▼ Rarely ... Often |
| Feel more tired than before | ▼ Rarely ... Often |
| A strong urge to sleep in the daytime | ▼ Rarely ... Often |
| Feel unwell | ▼ Rarely ... Often |
| Feel unrested | ▼ Rarely ... Often |
| Feel depressed | ▼ Rarely ... Often |
| Feel anxious | ▼ Rarely ... Often |
| Feel restless | ▼ Rarely ... Often |
| Feel irritable | ▼ Rarely ... Often |
| Feel apt to make errors | ▼ Rarely ... Often |
| Feel unfocused | ▼ Rarely ... Often |
| Feel unmotivated | ▼ Rarely ... Often |

**The following questions ask about your delivery history**.
  How many living children do you have (that you have birthed)?

- 1
- 2
- 3
- 4
- 5 or more

What is the date of birth of your **youngest** child?

|  | Month | Day | Year |
| --- | --- | --- | --- |
|  |  |  |  |
| Please Select: | ▼ January ... December | ▼ 1 ... 31 | ▼ 2016 ... 2020 |

What was the delivery type of your **youngest** child?

- Vaginal
- Vaginal assisted (forceps, suction etc.)
- C-Section
- Other- Please Specify: ________________________________________________

Are you currently breast feeding or pumping?

- Yes
- No

Display This Question:

If Are you currently breast feeding or pumping? = No

How many months did you breastfeed/pump for?

- I did not breastfeed
- 1-3 months
- 3-6 months
- 6-9 months
- 9-12 months
- 12-15 months
- 15-18 months
- 18-21 months
- 21-24 months
- > 24 months

| 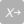 |
| --- |

The following questions ask about your core and pelvic health. 

Have you been diagnosed with a "Diastasis Recti" (separated abdominals)?

- Yes
- No
- Unsure
- Other- Please Specify: ________________________________________________

Do you leak any amount of **urine** when you cough, sneeze, change positions or run?

- Yes
- No

Do you leak **feces/gas** when you cough, sneeze, change positions or run?

- Yes
- No

**The following questions ask about your sleep**.
   In the past 7 days, how many total hours of sleep did you average each night?

________________________________________________________________

| 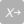 |
| --- |

On average in the past 7 days, how many interruptions in sleep do you have per night?

- 0
- 1
- 2
- 3
- 4
- 5
- More than 5

**The following questions ask about your PRIOR running injury history**.
  Have you **ever** had a running related injury that you went to see a health professional for? (not current injury if any)

- No
- One injury
- Two injuries
- Three injuries
- Four or more injuries

In which country do you currently reside?

▼ Afghanistan ... Zimbabwe

Display This Question:

If List of Countries = United States of America

In which state do you currently reside?

▼ Alabama ... I do not reside in the United States

| 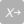 |
| --- |

What is your age (years)?

▼ 18 ... 50

What is your relationship status?

- Single
- Married
- Committed relationship/ partner
- Divorced
- Widowed
- Separated
- Never married/ No partner

End of Block: Country

Start of Block: Demographics Base/Universal

What is the highest level of school you have completed or the highest degree you have received?

- Less than high school degree
- High school graduate (high school diploma or equivalent including GED)
- Some post high school education but no degree
- Associate degree in technical college/ trade school (2-year)
- Bachelor's degree in university (4-year)
- Master's degree
- Doctoral degree
- Professional doctorate degree (JD, MD, DPT, DDS etc.)

Choose one or more races that you consider yourself to be:

- White
- Black or African American
- American Indian or Alaska Native
- Asian
- Native Hawaiian or Pacific Islander
- Prefer not to answer
- Other- Please Specify: ________________________________________________

End of Block: Demographics Base/Universal

**Please Note:**

**Questions related to data that has not been included in this manuscript have been deleted from this document.**
